# Epidemiology and outcomes of bloodstream infections in severe burn patients: a six-year retrospective study

**DOI:** 10.1186/s13756-021-00969-w

**Published:** 2021-06-30

**Authors:** Yangmin Hu, Danyang Li, Lingcheng Xu, Yuping Hu, Yiwen Sang, Gensheng Zhang, Haibin Dai

**Affiliations:** 1 Department of Pharmacy, Second Affiliated Hospital, Zhejiang University School of Medicine, Hangzhou, 310009 China; 2 Department of Critical Care Medicine, Second Affiliated Hospital, Zhejiang University School of Medicine, Hangzhou, 310009 China; 3 Department of Pharmacy, Hangzhou Third People’s Hospital, Hangzhou, 310009 China; 4 Department of Laboratory Medicine, Second Affiliated Hospital, Zhejiang University School of Medicine, Hangzhou, 310009 China

**Keywords:** Burns, Bloodstream infection, Multidrug resistance, Mortality

## Abstract

**Background:**

Infection is the leading cause of morbidity and mortality among burn patients, and bloodstream infection (BSI) is the most serious. This study aimed to evaluate the epidemiology and clinical outcomes of BSI in severe burn patients.

**Methods:**

Clinical variables of all patients admitted with severe burns (≥ 20% total body surface area, %TBSA) were analyzed retrospectively from January 2013 to December 2018 at a teaching hospital. The Kaplan–Meier method was utilized for plotting survival curves. Multivariate logistic regression and Cox regression model were also performed.

**Results:**

A total of 495 patients were evaluated, of whom 136 (27.5%) had a BSI. The median time from the patients being burned to BSI was 8 days. For BSI onset in these patients, 47.8% (65/136) occurred in the first week. The most frequently isolated causative organism was *A. baumannii* (22.7%), followed by methicillin-resistant *Staphylococcus aureus* (18.7%) and *K. pneumoniae* (18.2%), in patients with BSI. Multivariate logistic regression analysis showed that %TBSA (*p* = 0.023), mechanical ventilation (*p* = 0.019), central venous catheter (CVC) (*p* < 0.001) and hospital length of stay (27d vs 50d, *p* < 0.001) were independent risk factors associated with BSI. Cox regression model showed that acute kidney injury (HR, 12.26; 95% CI 2.31–64.98; *p* = 0.003) and septic shock (HR, 4.36; 95% CI 1.16–16.34; *p* = 0.031) were identified as independent predictors of 30-day mortality of BSI in burn patients.

**Conclusions:**

Multidrug resistant gram-negative bacteria were the main pathogens of BSI in severe burn patients. Accurate evaluation of risk factors for BSI and the mortality of BSI in severe burn patients may improve early appropriate management.

## Introduction

Infections are the leading cause of morbidity and mortality among burn patients [1, 2]. For patients with burns over 20% of the total body surface area (TBSA), the humoral and cellular immunity is also altered [3], and a disruption of the protective skin barrier and invasion of microorganisms into the burn eschar make the prevention and treatment of infection especially difficult [4]. One of the most troublesome infections that burn patients may develop is bloodstream infection (BSI) [5]. Burn patients are at a high risk of BSI because of multiple surgical procedures, the use of invasive devices and prolonged hospitalization [6]. Previous studies indicated that BSI was a predictor of poor outcome in burn patients [5, 7]. Therefore, it is essential to determine the characteristics of BSIs and find appropriate measures to prevent their occurrence.

Furthermore, due to rising antibiotic resistance worldwide, burn patients are at an increased risk of infections with multidrug-resistant (MDR) organisms, such as *methicillin-resistant Staphylococcus aureus* (MRSA), MDR *P. aeruginosa*, MDR *A. baumannii* and *K. pneumoniae* carbapenemase (KPC)-carrying strains [8, 9]. These patients who were at risk for these MDR organisms also rely on broader-spectrum antibiotic agents, which further drive resistance by sustained selective pressure. Therefore, it is necessary to implement appropriate antibiotic therapy protocols in burn patients. This study was conducted at a burn ward to evaluate the epidemiology and risk factors associated with 30-day mortality due to BSI in severe burn patients.

## Methods

### Study design and data collection

This study was conducted at the Second Affiliated Hospital of Zhejiang University, School of Medicine, which is a tertiary care hospital. Patients including pediatric and adult were admitted to the burn ward or the burn intensive care unit during the period from January 2013 to December 2018. The patients included had a burn that covered ≥ 20% TBSA with or without inhalation injury. Patients who were admitted to the hospital more than 24 h after burn or died within 48 h after admission were excluded. The data collected for burn patients included demographic characteristics, comorbidities, type and extent of burn, inhalation injury, mechanical ventilation, hospital length of stay, coinfections before BSI, acute kidney injury (AKI), treatment of BSI, and outcomes.

### Definitions

Severe burn was defined as those with burns greater than 20% TBSA with or without inhalation injury. Only patients admitted to this facility within 24 h of sustaining the burn wound were included in the study. Bloodstream infection (BSI) was defined as the isolation of bacteria or fungi from one or more blood cultures. If patients had more than one positive blood culture of same species during the same admission episode, only the first BSI episode was included. For coagulase-negative *Staphylococci*, *Corynebacterium* spp*.*, *Bacillus* and other common skin contaminants, at least two separate blood cultures with the same organisms were required to be positive for these to be considered as pathogens [10]. The definitions of skin infection, pneumonia, central venous catheter (CVC) and urinary tract infection (UTI) followed the relevant guidelines [10, 11]. Empirical antimicrobial therapy was defined as a treatment administered to patients suspected of having bacteremia before microbiological results were available. Definitive therapy was defined as the treatment of antibiotics administered after obtaining susceptibility results. Appropriate therapy was defined as receiving at least one active antibiotic against the isolated pathogen within 48 h, according to the results of species identification and susceptibility test.

### Microbiology

Blood culture samples consisted of an aerobic bottle and an anaerobic bottle, and the two sets of samples were collected at two puncture sites. The blood culture bottles were incubated at approximately 37℃ for up to 5 days in the semi-automated continuous monitoring blood culture system BacT/ALERT 3D (BioMérieux, France). Gram stain and subcultures on solid media were performed from positive blood cultures. Microbiology species identification and susceptibility testing were performed in the clinical laboratory by the VITEK 2 system (bioMérieux, France). MDR was defined as acquired non-susceptibility to at least one agent in three or more antimicrobial categories [12].

### Statistical analysis

Statistical analysis was performed using SPSS version 25.0. The chi-square test and Fisher’s exact test were used to compare categorical variables. Continuous variables were described as the median and interquartile range (IQR), and differences were identified using the Student *T* test or the Mann–Whitney *U* test. Potential risk factors for BSI or mortality were evaluated by using univariate analysis, and those with a *p* value < 0.1 were further analyzed with multivariate logistic regression or Cox regression model to detect which factors were independently associated with BSI or mortality. The Kaplan–Meier method was utilized for plotting survival curves, and differences were compared using the log-rank test. All p values were two-tailed, and a *p* value < 0.05 was considered statistically significant.

## Results

During the six-year study period, a total of 495 patients were evaluated, of whom 371 (74.9%) were male. Their ages were between 13 and 99 years old (median 46 years, [IQR] 36–56 years). Most patients (n = 399, 80.6%) were flame injuries. Of these patients, 88.5% (n = 438) had accompanying third degree burns, and 42.8% (n = 212) had inhalation injury. The median length of hospitalization was 35 days (IQR, 17–52 days) (Table 1).

**Table 1:** Risk factors for BSI of the patients with burns over 20% TBSA

Characteristic, n (%)	Total (n = 495)	Non-BSI (n = 359)	BSI (n = 136)	Univariate analysis *p* value	Multivariate logistic regression analysis	
					OR (95% CI)	*p* value
Male	371 (74.9)	271 (75.5)	100 (73.5)	0.654	–	–
Age (median, IQR)	46 [36,56]	46 [35,56]	47 [37,56]	0.728	–	–
Comorbidities	121 (24.4)	87 (24.2)	34 (25.0)	0.859	–	–
Type of burn						
Flame	399 (80.6)	283 (78.8)	116 (85.3)	0.104	–	–
Chemical	26 (5.3)	21 (5.8)	5 (3.7)	0.333	–	–
Hyperthermia liquid	49 (9.9)	37 (10.3)	12 (8.8)	0.622	–	–
Other	21 (4.2)	18 (5.0)	3 (2.2)	0.166	–	–
%TBSA						0.023
20–39	212 (42.8)	185 (51.5)	27 (19.9)	˂0.001	–	–
40–59	130 (26.3)	98 (27.3)	32 (23.5)		1.08 (0.55–2.09)	0.828
60–79	64 (12.9)	31 (8.6)	33 (24.3)		2.53 (1.21–5.28)	0.014
≥ 80	89 (18.0)	45 (12.5)	44 (32.4)		2.24 (1.01–4.97)	0.048
Accompanying third degree burns	438 (88.5)	312 (86.9)	126 (92.6)	0.078	0.70 (0.29–1.69)	0.423
Inhalation injury	212 (42.8)	132 (36.8)	80 (58.8)	˂0.001	0.85 (0.51–1.44)	0.547
Mechanical ventilation	178 (36.0)	96 (26.7)	82 (60.3)	˂0.001	1.97 (1.12–3.47)	0.019
CVC	352 (71.1)	220 (61.3)	132 (97.1)	˂0.001	9.10 (3.06–27.04)	˂0.001
AKI	56 (11.3)	34 (9.5)	22 (16.2)	0.038	0.80 (0.38–1.67)	0.550
Hospital length of stay, days (median, IQR)	35 [17,52]	27 [15,44]	50 [33,67]	˂0.001	1.02 (1.01–1.03)	˂0.001

*BSI* bloodstream infection, *IQR* interquartile range, *TBSA* total body surface area, *CVC* Central venous catheter, *AKI* acute kidney injury

There were 136 (27.5%) patients who had a BSI during hospitalization. Univariate analysis showed that %TBSA (*p* < 0.001), inhalation injury (*p* < 0.001), mechanical ventilation ((*p* < 0.001), CVC (*p* < 0.001), AKI (*p* = 0.038) and hospital length of stay (*p* < 0.001) were associated with BSI. The results of multivariate logistic regression analysis showed that %TBSA (*p* = 0.023), mechanical ventilation (*p* = 0.019), CVC (*p* < 0.001) and hospital length of stay (*p* < 0.001) were independent risk factors associated with BSI (Table 1). The median time from the patients being burned to the first episode of BSI was 8 days (IQR, 5–16 days). For BSI onset in these patients, 47.8% (65/136) occurred in the first week (Table 2).

**Table 2:** Duration from admission to the first episode of BSI in burn patients

Time after admission	Total (n = 136)	Death (n = 25)	Survival (n = 111)
1st week	65 (47.8)	11 (44.0)	54 (48.6)
2nd week	38 (27.9)	9 (36.0)	29 (26.1)
3rd week	19 (14.0)	4 (16.0)	15 (13.5)
≥ 4th week	14 (10.3)	1 (4.0)	13 (11.7)
Duration from admission to bacteremia (days) (median, IQR)	8 [5–16]	10 [5–13.5]	8 [5–17]

Overall, there were 225 isolates from the bloodstream of 136 patients. Among them, 45.6% (62/136) patients had more than one positive culture, so the number of cultures was higher than the number of patients. The most frequently isolated causative organism was *A. baumannii* (22.7%), followed by MRSA (18.7%) and *K. pneumoniae* (18.2%) in patients with BSI (Table 3)*.* Gram-negative bacteria were more common than Gram-positive bacteria. In these Gram-negative bacteria, most of them showed a MDR phenotype. Most (95%) *A. baumannii* isolates were resistant to ceftazidime, cefepime, ciprofloxacin, piperacillin-tazobactam and carbapenems. The resistance frequencies of *K. pneumoniae* to amikacin and tigecycline were 52.8% and 24.0% respectively. All *A. baumannii, K. pneumoniae* and *P. aeruginosa* isolates were susceptible to polymyxin B. (Table 4).

**Table 3:** Causative organisms in burn patients with BSI

Pathogens	n (n = 225) (%)
*A. baumannii*	51 (22.7)
*K. pneumoniae*	41 (18.2)
*P. aeruginosa*	22 (9.8)
MRSA	42 (18.7)
MRCNS	23 (10.2)
*Enterococcus* species	15 (6.7)
*Stenotrophomonas maltophilia*	7 (3.1)
*Escherichia coli*	7 (3.1)
*Enterobacter cloacae*	5 (2.2)
*Proteus mirabilis*	2 (0.9)
*Burkholderia cepacia*	2 (0.9)
*Morganella morganii*	1 (0.4)
*Ralstonia pickettii*	1 (0.4)
*Candida* species	6 (2.7)

*MRSA* methicillin-resistant *Staphylococcus aureus*, *MRCNS* methicillin-resistant *Coagulase negative staphylococci*

**Table 4:** Antibiotic resistance in Gram-negative isolates of BSI

Antibiotics	*A. baumannii, *% resistant (n = 51)	*K. pneumoniae*, % resistant (n = 41)	*P. aeruginosa*, % resistant (n = 22)
Amikacin	75.0	52.8	45.0
Ceftazidime	95.0	88.9	30.0
Cefepime	95.0	80.6	65.0
Ciprofloxacin	95.0	86.1	50.0
Levofloxacin	72.5	80.6	55.0
Imipenem	95.0	77.8	75.0
Meropenem	95.0	77.8	75.0
Cefoperazone-Sulbactam	72.5	88.9	80.0
Piperacillin-Tazobactam	95.0	80.6	80.0
Tigecycline	11.4 (n = 45)	24.0 (n = 29)	–
Polymyxin B	0.0 (n = 21)	0.0 (n = 18)	0.0 (n = 9)

**Fig. 1:**
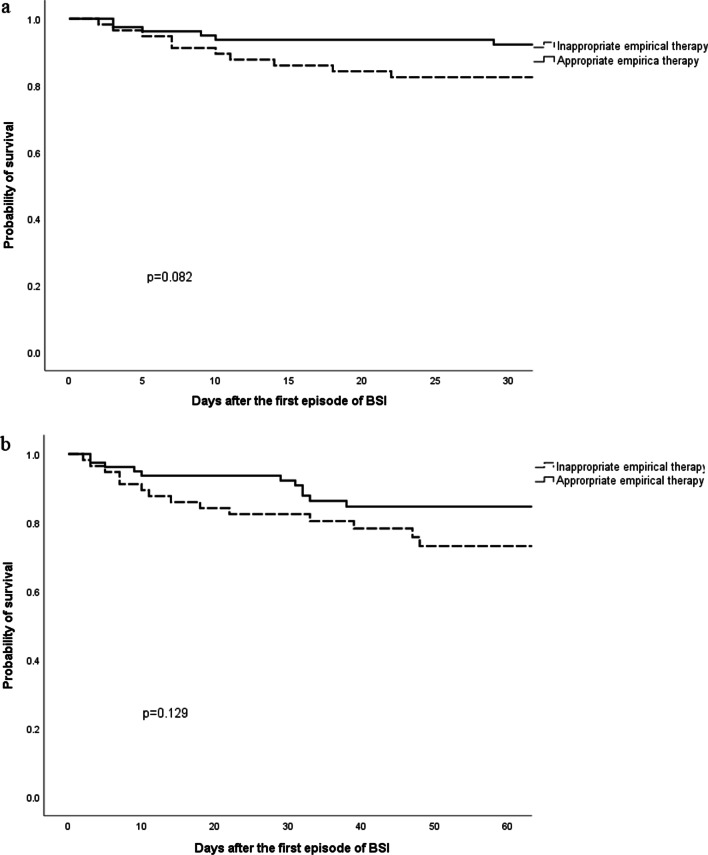
Kaplan–Meier curves of 30-day mortality (**a**) and 60-day mortality (**b**) according empiric therapy for BSI in burn patients

Among the 136 patients with BSI, 58.1% (n = 79) patients received appropriate and 41.9% (n = 57) received inappropriate, empirical antimicrobial therapy. A significant difference was found in skin coinfection before BSI (*p* = 0.021), multiple organ dysfunction syndrome (MODS) (*p* = 0.043) and inappropriate definitive therapy (*p* = 0.001) between the two groups (Table 5). The 30-day and 60-day mortality were lower in the appropriate empirical antimicrobial therapy group than in the inappropriate empirical antimicrobial therapy group (7.6% vs 17.5%, *p* = 0.076, and 13.9% vs. 24.6%, *p* = 0.114, respectively), but there was no significant difference between the two groups. The Kaplan–Meier curves also showed no significant difference in the 30-day (*p* = 0.082) and 60-day mortality (*p* = 0.129) between the two groups (Fig. 1).

**Table 5:** Differences of characteristics of BSI burn patients receiving appropriate and inappropriate empirical antimicrobial therapy

Variable	Empirical antimicrobial therapy		
	Appropriate n = 79	Inappropriate n = 57	*p*-value
Male	59 (74.7)	41 (71.9)	0.719
Age (years) (median, IQR)	47 [34,54]	46 [38,56]	0.601
Comorbidities	19 (24.1)	15 (26.3)	0.763
Type of burn			
Flame	70 (88.6)	46 (80.7)	0.199
Chemical	2 (2.5)	3 (5.3)	0.404
Hyperthermia liquid	5 (6.3)	7 (12.3)	0.227
Other	2 (2.5)	1 (1.8)	0.761
%TBSA			
20–39	16 (20.3)	11 (19.3)	0.341
40–59	19 (24.1)	13 (22.8)	
60–79	23 (29.1)	10 (17.5)	
≥ 80	21 (26.6)	23 (40.4)	
Accompanying third degree burns	73 (92.4)	53 (93.0)	0.899
Inhalation injury	51 (64.6)	29 (50.9)	0.110
Mechanical ventilation	48 (60.8)	34 (59.6)	0.896
Coinfections before bacteremia			
Skin infection	65 (82.3)	37 (64.9)	0.021
Pneumonia	38 (48.1)	19 (33.3)	0.085
CVC infection	24 (30.4)	14 (24.6)	0.456
UTI	3 (3.8)	3 (5.3)	0.681
AKI	9 (11.4)	13 (22.8)	0.074
Septic shock	9 (11.4)	9 (15.8)	0.455
MODS	7 (8.9)	12 (21.1)	0.043
Hospital length of stay, days (median, IQR)	51 [34,68]	47 [30,66]	0.642
Inappropriate definitive antimicrobial therapy	3 (3.8)	13 (22.8)	0.001
Outcomes			
30-day mortality	6 (7.6)	10 (17.5)	0.076
60-day mortality	11 (13.9)	14 (24.6)	0.114

*BSI* bloodstream infection, *IQR* interquartile range, *TBSA* total body surface area, *CVC* central venous catheter, *UTI* urinary tract infection, *AKI* acute kidney injury, *MODS* multiple organ dysfunction syndrome

By entering the %TBSA (*p* = 0.001), mechanical ventilation (*p* = 0.021), AKI (*p* < 0.001), septic shock (*p* < 0.001), inappropriate empirical antimicrobial therapy (*p* = 0.093) and inappropriate definitive antimicrobial therapy (*p* = 0.010) in the Cox regression model, AKI (HR, 12.26; 95% CI 2.31–64.98; *p* = 0.003) and septic shock (HR, 4.36; 95% CI 1.16–16.34; *p* = 0.031) were identified as independent predictors of 30-day mortality of BSI in burn patients (Table 6).

**Table 6:** Univariate and multivariate Cox regression analyses of variables associated with 30-day mortality of BSI in burn patients

Variable	Crude analysis		Adjusted analysis	
	HR (95%CI)	*p* value	HR (95%CI)	*p* value
Male	0.70 (0.20–2.47)	0.583		
Age	1.00 (0.97–1.04)	0.856		
Comorbidities	0.72 (0.21–2.53)	0.609		
Type of burn (Flame)	0.52 (0.17–1.60)	0.253		
≥ 80% TBSA	0.14 (0.05–0.44)	0.001	0.86 (0.22–3.38)	0.861
Accompanying third degree burns	22.7 (0.01,7204.37)	0.448		
Inhalation injury	1.55 (0.54–4.46)	0.417		
Mechanical ventilation	10.77 (1.42–81.54)	0.021	4.14 (0.41–41.53)	0.228
Coinfections before bacteremia				
Skin infection	0.72 (0.25–2.08)	0.546		
Pneumonia	1.40 (0.53–3.72)	0.503		
CVC infection	0.36 (0.08–1.57)	0.171		
UTI	1.43 (0.19–10.84)	0.728		
AKI	42.85 (12.10–151.78)	< 0.001	12.26 (2.31–64.98)	0.003
Septic shock	36.98 (12.79–106.87)	< 0.001	4.36 (1.16–16.34)	0.031
Inappropriate empirical antimicrobial therapy	0.42 (0.15–1.16)	0.093	0.56 (0.17–1.89)	0.351
Inappropriate definitive antimicrobial therapy	0.25 (0.09–0.72)	0.010	0.52 (0.12–2.19)	0.371

*HR* hazard ratio, *CI* confidence interval, *TBSA* total body surface area, *CVC* central venous catheter, *UTI* urinary tract infection, *AKI* acute kidney injury

## Discussion

Invasive infection, especially BSI, is now the chief reason for morbidity and mortality after burn injury [1, 13]. Patel BM et al. found that 4% of examined burn patients developed a BSI [5]. However, our study found that 27.5% of the burn patients developed a BSI, probably because we only included serious burn patients with a burn extent ≥ 20% TBSA. We found that the median time from the patients being burned to BSI was 8 days and that 47.8% of BSIs occurred in the first week. This finding was similar to the results found in other studies that demonstrated the median time from burn to BSI to be 7 days [5] or 6 days [14]. Thus, it is important to control infection in the first week after the hospitalization of burn patients.

Our study showed that %TBSA, mechanical ventilation, CVC use and hospital length of stay were independent risk factors associated with BSI. Burn is independently associated with the development of nosocomial BSI [15], and the incidence of BSI increases with %TBSA [4, 16]. However, burn wounds are not the only point of entrance for pathogens that cause BSI [7]. Other factors, such as CVC use, other infections and receipt of mechanical ventilation, should be considered to be associated with BSI [17]. It has been identified that CVC is the most frequent cause of nosocomial BSI [14, 18]. Mechanical ventilation and prolonged hospital stay increase the likelihood of exposure to nosocomial infections including BSI, which complicates the treatment of the patients [19, 20]. The implementation of the various active prevention strategies is essential to reduce BSI and improves the prognosis of burn patients. Intervention measures should include optimal care, strict hand hygiene, reduction of mechanical ventilation duration, antimicrobial therapies and continuous monitoring of infection development [21, 22].

Some studies found that Gram-positive organisms, particularly *Staphylococcus aureus*, were more commonly associated with BSI than Gram-negative organisms [5, 23], whereas other studies showed that *P. aeruginosa* was the most prevalently isolated species from burn patients [24, 25]. However, we found that *A. baumannii* was the most common organism isolated from burn patients with BSI, followed by MRSA and *K. pneumoniae.* At another Chinese burn institute, the resistance frequencies of *A. baumannii* isolated from the BSI of burn patients to imipenem and meropenem were 94% and 91%, respectively [26], which are close to our result, 95%. MDR organism infections resulted in a increased length of hospitalization, elevated need for mechanical ventilation and prolonged duration of antibiotic treatment [8]. These data emphasized that additional attention should be paid to monitoring the local microbiology and antimicrobial susceptibility test reports for medical institutions to prevent further distribution.

One of the major challenges in treating BSI is bacterial resistance to antibiotics, and appropriate therapy is associated with protective effect on mortality [27]. Even if there is no proven infection, antibiotic therapy is often used as an empirical measure for burn patients according to the symptoms and signs of infection [28], or due to other infections such as skin infection or pneumonia before BSI. In our study, 58.1% (n = 79) of severe burns patients with BSI received appropriate empirical antimicrobial therapy. MODS in the appropriate empirical antimicrobial therapy group was significantly lower than that in the inappropriate empirical antimicrobial therapy group. For burn patients, MODS was recognized as a leading cause of death [8]. However, the mortality in patients who did receive appropriate empirical therapy was not better than the mortality in patients who received inappropriate empirical therapy in our study, though a possible trend was noted. It was possible that the sample size in our study was too small to demonstrate whether inappropriate therapy was associated with mortality. Most BSIs treated inappropriately were caused by MDR Gram-negative organisms.

Severe infections causing septic shock occur frequently in burn patients and AKI is also a common complication in these patients. In this study, both septic shock and AKI were shown to be the risk factors for 30-day mortality of BSI in severe burn patients. Other works support our finding that the AKI and septic shock significantly increased the mortality in burn patients [29–31].

The current study has some potential limitations. First, the cultures from skin lesions were considered as being episodes of skin coinfection, but the organisms isolated from these cultures may have been colonisers and were not necessarily causing infection. Second, coinfection was quite common during hospitalization of patients, so we could not identify which specific organism caused mortality with BSI. Third, we did not analyze the impact of MDR organisms on mortality or other clinical outcomes because most of the isolated bacteria were MDR; however, this effect can be a focus of future research. Finally, we cannot exclude that confounding factors were potentially associated with the mortality of BSI in burn patients, although we had considered as many factors as possible.

## Conclusions

This study demonstrated that MDR gram-negative bacteria were the main pathogens of BSI in severe burn patients. Mechanical ventilation, %TBSA, CVC and hospital length of stay were independent risk factors associated with BSI, and AKI and septic shock were identified as the independent predictors of 30-day mortality of BSI in burn patients. Accurate evaluation of risk factors for BSI and the mortality of BSI in severe burn patients may improve early appropriate management. Additional studies and greater sample size are needed to further investigate the potential impact of appropriate antibiotic treatment on mortality.

## Data Availability

All data generated or analyzed during this study were included in this manuscript.
